# Protein tyrosine phosphatase 4A3 (PTP4A3/PRL-3) drives migration and progression of T-cell acute lymphoblastic leukemia in vitro and in vivo

**DOI:** 10.1038/s41389-020-0192-5

**Published:** 2020-01-30

**Authors:** M. Wei, M. G. Haney, D. R. Rivas, J. S. Blackburn

**Affiliations:** 1 Department of Molecular and Cellular Biochemistry, University of Kentucky, Lexington, KY 4053 USA; 2 Markey Cancer Center, Lexington, KY 40536 USA

**Keywords:** Oncogenes, Acute lymphocytic leukaemia, Oncogenes, Acute lymphocytic leukaemia

## Abstract

T-cell acute lymphoblastic leukemia (T-ALL) is an aggressive blood cancer. There are no immunotherapies and few molecularly targeted therapeutics available for treatment of this malignancy. The identification and characterization of genes and pathways that drive T-ALL progression are critical for the development of new therapies for T-ALL. Here, we determined that the protein tyrosine phosphatase 4A3 (PTP4A3 or PRL-3) plays a critical role in T-ALL initiation and progression by promoting leukemia cell migration. PRL-3 is highly expressed in patient T-ALL samples at both the mRNA and protein levels compared to normal lymphocytes. Knock-down of PRL-3 expression using short-hairpin RNA (shRNA) in human T-ALL cell lines significantly impeded T-ALL cell migration capacity in vitro and reduced their ability to engraft and proliferate in vivo in xenograft mouse models. Additionally, PRL-3 overexpression in a *Myc*-induced zebrafish T-ALL model significantly accelerated disease onset and shortened the time needed for cells to enter blood circulation. Reverse-phase protein array (RPPA) and gene set enrichment analysis (GSEA) revealed that the SRC signaling pathway is affected by PRL-3. Immunoblot analyses validated that manipulation of PRL-3 expression in T-ALL cells affected the SRC signaling pathway, which is directly involved in cell migration, although Src was not a direct substrate of PRL-3. More importantly, T-ALL cell growth and migration were inhibited by small molecule inhibition of PRL-3, suggesting that PRL-3 has potential as a therapeutic target in T-ALL. Taken together, our study identifies PRL-3 as an oncogenic driver in T-ALL both in vitro and in vivo and provides a strong rationale for targeted therapies that interfere with PRL-3 function.

## Introduction

T-cell acute lymphoblastic leukemia (T-ALL) is an aggressive hematologic malignancy, representing 10–15% of pediatric and 25% of adult ALL cases^1^. The treatment of T-ALL lags behind that of B-cell ALL (B-ALL) and other leukemia subtypes in regard to both availability of immunotherapies and the development of molecular targeted therapies^2^. Additionally, relapsed T-ALL remains a major clinical concern, with less than 30% of children and 10% of adults surviving relapse, and current intensive chemotherapy regimens for T-ALL have long-term adverse effects in patients^1,3–5^. More effective and selective treatment strategies are critically needed for T-ALL. The development of novel therapeutics requires the identification and characterization of targetable drivers of T-ALL progression.

Protein phosphatases cooperate with kinases to precisely maintain appropriate protein phosphorylation and have important roles in modulating the strength and duration of signaling events, critical for normal cellular functions. Abnormal protein phosphorylation is a common feature in cancer and disease. While kinase inhibitors have achieved significant success in clinic^6^, phosphatases are underexplored as drug targets^7,8^, largely due to the misconception that phosphatases function primarily as tumor suppressors, as well as the challenges in developing specific phosphatase inhibitors. To date, more than 30 potentially oncogenic phosphatases have been identified, and are being explored as drug targets in cancer therapy^9^.

Protein tyrosine phosphatase 4A3 (PTP4A3), also known as phosphatase of regenerating liver 3 (PRL-3), is an oncogenic phosphatase that has received significant attention as a potential therapeutic target in a variety of cancers^7,8,10^. PRL-3 is highly expressed in ~80% of 151 human tumor tissue samples across 11 tumor types, including liver, lung, colon, breast, stomach, thyroid, pancreas, kidney, bladder, and prostate cancer^10^, and PRL-3 has been extensively reported as a biomarker of tumor progression and metastasis in breast^11^, colon^12,13^, gastric^14^, brain^15^, and prostate^16^ cancers. Elevated PRL-3 correlates with reduced survival in patients with breast^17^, gastric^14^, ovarian^18^, and liver^19^ cancers and in acute myelogenous leukemia (AML)^20,21^. More importantly, the causative role of PRL-3 in solid tumors has been functionally demonstrated by overexpression and knock-down of PRL-3 in normal or cancer cells. For example, ectopic expression of PRL-3 in human melanoma, breast, lung, and colorectal cancer cells has been reported to increase cell motility, migration, invasion, and proliferation in vitro and to accelerate tumor formation, progression, and metastasis in vivo^22–25^. Similarly, knock-down of PRL-3 expression using short-hairpin RNA (shRNA) led to decreased cell proliferation, adhesion, migration, and invasion in a range of solid tumors in vitro and inhibited primary tumor proliferation and invasion in vivo in colorectal, gastric and ovarian cancers and in melanoma, ultimately improving the prognosis and life span of mice^26–29^.

Given the proven role of PRL-3 in solid tumor malignancies, efforts have been made to develop specific PRL-3 inhibitors, including JMS-053^30^, Compound 43 and its analogs^31^, and Analog 3^32^, all of which target the entire PRL family (PRL-1, −2, and −3). In addition, a humanized PRL-3 antibody has been developed that specifically targets PRL-3 over other family members^10,33^. These efforts suggest that PRL-3 is a feasible therapeutic target in cancer.

The role of PRL-3 in leukemia is less well defined, and its contribution to T-ALL progression has not been reported. Here, we demonstrate that PRL-3 plays a role in T-ALL development and migration both in vitro and in vivo in mice and zebrafish, and we provide a mechanism by which PRL-3 may function as an oncogene in ALL via modulation of the SRC signaling pathway to promote T-ALL migration. Taken together, our study identifies a critical role of PRL-3 in T-ALL onset and progression both in vitro and in vivo and suggests that PRL-3 may be a targetable oncogenic driver in T-ALL.

## PRL-3 is highly expressed in T-ALL patient samples and T-ALL cell lines

**Fig. 1:**
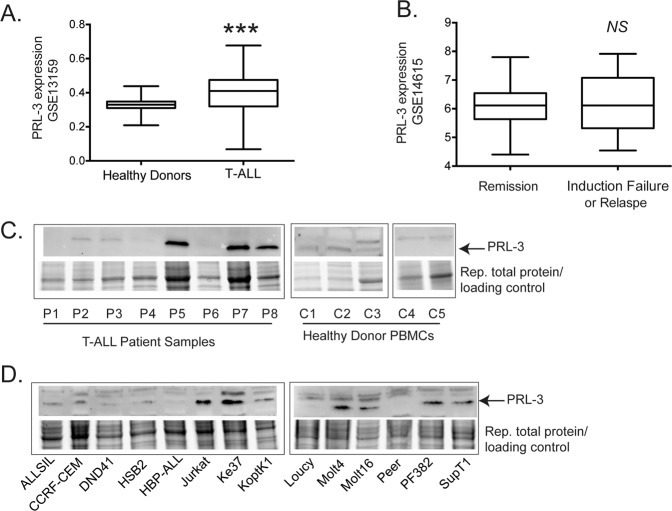
PRL-3 is highly expressed in a majority of human T-ALL. **a** Microarray expression analysis of GSE13159 comparing bone marrow samples from healthy donors (*n* = 72) and T-ALL patients (*n* = 174), ***p = 6.8e−10. **b** Analysis of GSE14615 comparing PRL-3 expression between bone marrow samples from T-ALL patients achieving remission and patients with induction failure. NS = not significant. Representative western blot analysis of (**c**) primary patient T-ALL and PBMCs, and (**d**) human T-ALL cell lines, showing PRL-3 expression. The total protein loaded in each sample was used as loading control instead of housekeeping protein, with a band of ~50 kD chosen as a representative image in the figure.

Analysis of bone marrow aspirate from T-ALL patients showed that PRL-3 mRNA expression was significantly higher in primary T-ALL (*n* = 174) compared to healthy donor samples (*n* = 72, GSE13159, *p* = 6.8e−10, Fig. 1a), although there was no significant difference found in PRL-3 expression between patients who achieved complete remission (*n* = 29) versus those that relapsed (*n* = 11) or suffered induction failure (*n* = 7, GSE14615, Fig. 1b). However, the sample size was relatively small in the latter study, and further investigation is warranted to determine whether PRL-3 expression may be a predictor of T-ALL treatment failure. Analysis of other PRL family members showed that PRL-1 expression was significantly lower in primary T-ALL patient samples compared to healthy bone marrow, while PRL-2 expression is significantly higher (Supplemental Fig. 1).

Western blot showed 3 out of 8 T-ALL patient peripheral blood mononuclear cell (PBMC) samples expressed very high PRL-3, while it was detected at low levels, if at all, in PBMCs from five healthy donors (Fig. 1c). Additionally, PRL-3 protein was expressed at varying levels across 14 T-ALL cell lines (Fig. 1d). Interestingly, PRL-3 expression in the same cell line fluctuated notably across independent assays (Supplemental Fig. 2), although we did not find the expression level to be related to cell density or serum deprivation. Consistent with gene expression datasets, PRL-1 was not detected across T-ALL cell lines, while PRL-2 protein was expressed in most T-ALL cell lines examined (Supplemental Fig. 3).

## PRL-3 knock-down in T-ALL cell lines inhibits cell migration in vitro and engraftment in a xenograft mouse model

**Fig. 2:**
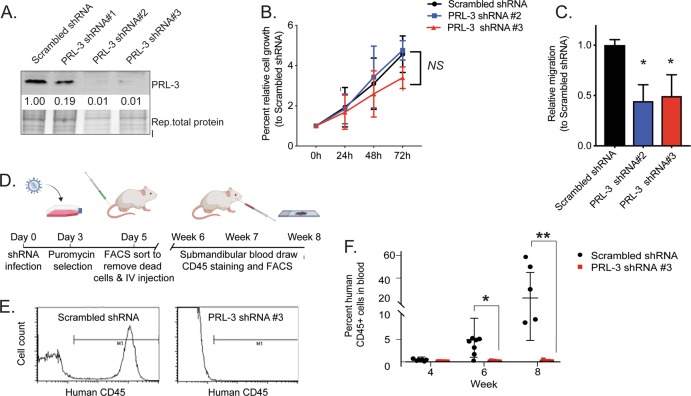
PRL-3 knock-down inhibits cell migration and T-ALL engraftment in a xenograft mouse model. **a** Representative western blot analysis figure showing PRL-3 protein expression in Jurkat T-ALL cells 4d post-infection with lentivirus carrying shRNA. Numbers represent relative expression of PRL-3 protein, normalized to total protein loaded and compared to scrambled (SCR) control. **b** Cells infected with SCR or PRL-3 knock-down shRNA were cultured in media 4 days post-infection with 5 μg/ml puromycin for 72 additional hours. Cell growth was determined by Cell Titer-Glo assay and normalized to the readout of day 0, and shows no difference between groups. Data shown are the average of three independent experiments, done in triplicate, NS = not significant. **c** Knock-down of PRL-3 in Jurkat cell line reduced migration towards a serum stimulus more than 50%. Migration was normalized to the cells infected by SCR shRNA, *p* < 0.05 compared to SCR control, *p < 0.05. **d** Schematic diagram of the xenotransplantation assay. **e** Representative flow cytometry analysis of submandibular blood sample after human CD45 staining. **f** Quantification of human CD45 staining of blood from mice at week 4,6, and 8 after transplantation. Each dot represents one mouse, the horizontal line represents the mean value, and the standard deviation is shown, *p < 0.01 and **p < 0.001 compared to shRNA control xenografted mice.

In order to define the role of PRL-3 in T-ALL, we used shRNAs to knock-down PRL-3 expression in Jurkat cells, a T-ALL line with high endogenous PRL-3 expression. Western blot analysis of samples collected four days after lentiviral infection of shRNA constructs showed that PRL-3 was successfully knocked-down by shRNA constructs #2 and #3 compared to scrambled (SCR) control shRNA (Fig. 2a). Expression of the other PRLs did not increase to compensate for PRL-3 loss (Supplemental Fig. 4A). Interestingly, despite puromycin selection of the shRNA construct, PRL-3 expression levels recovered over time (Supplemental Fig. 4B), suggesting that cells with higher PRL-3 expression may outcompete those with stronger knock-down.

PRL-3 knock-down did not negatively impact cell growth (Fig. 2b), however, silencing PRL-3 expression significantly reduced cell migration by approximately 50% (*p* < 0.02, Fig. 2c). Similarly, when we overexpressed PRL-3 in Jurkat and HBP-ALL cells (Supplemental Fig. 5A), we found no significant difference in cell growth (Supplemental Fig. 5B), but significantly enhanced migratory capability compared to control (*p* ≤ 0.009, Supplemental Fig. 5C). Together, these data suggest that PRL-3 plays an important role in regulating cell migration, but not proliferation, in T-ALL cells in vitro.

In order to determine whether silencing PRL-3 expression in human T-ALL affects its oncogenic ability in vivo, Jurkat cells expressing a scrambled shRNA or a shRNA targeting PRL-3 were injected intravenously into immune-compromised mice. At 4, 6, and 8 weeks after transplantation, blood samples were collected and stained with anti-human CD45 (Fig. 2d). Flow cytometry showed no human CD45-positive cells in the circulation of mice injected with PRL-3 knock-down Jurkat cells, while mice injected with Jurkat expressing scrambled shRNA cells showed increasing numbers of CD45 positive cells in the blood each week (Fig. 2e, f). Three mice harboring scrambled shRNA expressing T-ALL had to be euthanized before the 8-week time point due to mobility issues likely caused by T-ALL infiltration into the spine or central nervous system, while mice with PRL-3 knock-down remained healthy throughout the duration of the study. The survival of mice xenografted with PRL-3 knock-down T-ALL cells may be due to decreased ability of the cells to engraft and/or circulate, and further studies are needed to differentiate between these possibilities.

## PRL-3 enhances T-ALL onset in a zebrafish model

**Fig. 3:**
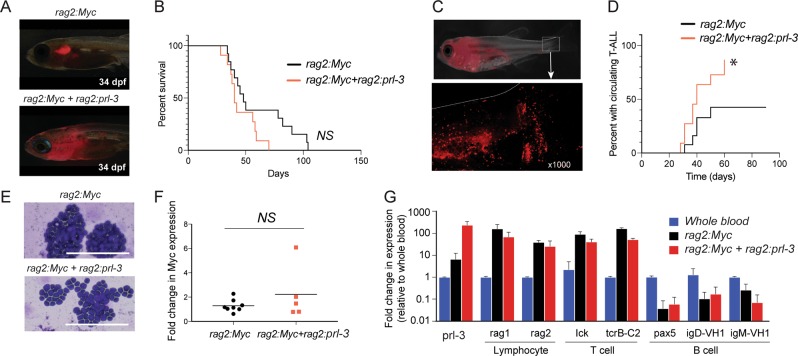
PRL-3 enhances circulation of leukemia cells in a zebrafish T-ALL model. **a** Representative images of transient transgenic zebrafish expressing *rag2:Myc* *+* *rag2:mCherry* (*n* = 11) or *rag2:Myc* *+* *rag2:mCherry* *+* *rag2:prl-3* (*n* = 6) at 34 days post-fertilization (dpf). **b** Kaplan–Meier analysis of time (days) percent survival (>70% of animal is mCherry-positive). **c** Representative *rag2:Myc* *+* *rag2:mCherry* *+* *rag2:prl-3* animal, showing circulating mCherry + leukemia cells within the tail fin. **d** Kaplan–Meier analysis of time (days) for each T-ALL to be visualized in circulation, * *p* = 0.049. **e** Representative images of May-Gunwald Giemsa staining of blood samples from fish from each leukemia type. Scale bar = 100 μm. **f** Realtime RT-PCR analysis of *Myc* expression between *rag2:Myc* *+* *rag2:mCherry* (*n* = 8) and *rag2:Myc* *+* *rag2:mCherry* *+* *rag2:prl-3* (*n* = 5). Each point represents one fish sample. NS = not significant. **g** Realtime RT-PCR analysis of lymphocyte, T-cell, and B-cell specific genes. Bars are the average expression of three samples per group.

The elevated expression of PRL-3 in T-ALL patient samples and its role in promoting migration in T-ALL cell lines suggests it may play an oncogenic role in T-ALL. We used a zebrafish *Myc*-induced T-ALL model^34,35^ to assess the role of PRL-3 in T-ALL onset and progression. Zebrafish *prl-3* has 88% homology to human *PRL-3* with conservation of critical domains^36^. One-cell stage zebrafish embryos were injected with plasmids containing *rag2:Myc* with *rag2:mCherry*, and with or without *rag2:prl-3*; the rag2 promoter drives gene expression in lymphocytes. T-ALL developed in zebrafish from the thymus and expanded into local tissues before entering the circulation. Fish were monitored for leukemia growth by quantifying the percent mCherry-positive cells within the body of the animal; >70% mCherry-positive was considered leukemic. Zebrafish T-ALL that expressed *prl-3* consistently expanded from the thymus into surrounding tissues earlier than T-ALL expressing *Myc* alone (Fig. 3a), although there was no significant difference in time to full leukemia onset between the groups (Fig. 3b). Because the T-ALL cells were fluorescently labeled, we were also able to determine the time at which leukemia cells begin to circulate by visualizing cells within the vasculature in the tail fin (Fig. 3c, Supplemental Videos 1 and 2). While more than half of animals with T-ALL in the *Myc*-expressing group never developed circulating disease by >100d, more than 80% of the *Myc* *+* *prl-3* expressing T-ALLs were circulating at a median time point of 42d, *p* = 0.05 (Fig. 3d).

The lymphoblasts were morphologically similar between groups (Fig. 3e), and there was no significant difference in Myc expression between *Myc* and *Myc* *+* *prl-3* T-ALL samples (Fig. 3f). Gene expression analyses indicated that both the *rag2:Myc* and *rag2:Myc* + *rag2:prl-3* leukemias expressed the lymphocyte specific genes *rag1* and *rag2* and the T-cell genes *lck* and *tcrB*, but not B-cell related genes *igD* or *igM*, indicating all leukemias generated were of T-cell origin. We verified that the *rag2:Myc* *+* *rag2:prl-3* leukemias expressed >10-fold higher levels of PRL-3 than the *Myc* control group (Fig. 3g). Interestingly, endogenous *prl-3* expression was also significantly higher in the *rag2:myc* T-ALL than normal zebrafish blood cells, suggesting that PRL-3 may be an important collaborating oncogene in T-ALL development. Taken together, these data suggest that PRL-3 can play an important role in T-ALL onset and progression in vivo, likely by enhancing migration into local tissues and contributing to the ability of the cells to enter circulation.

## PRL-3 modulates SRC pathway signaling to promote T-ALL migration

**Fig. 4:**
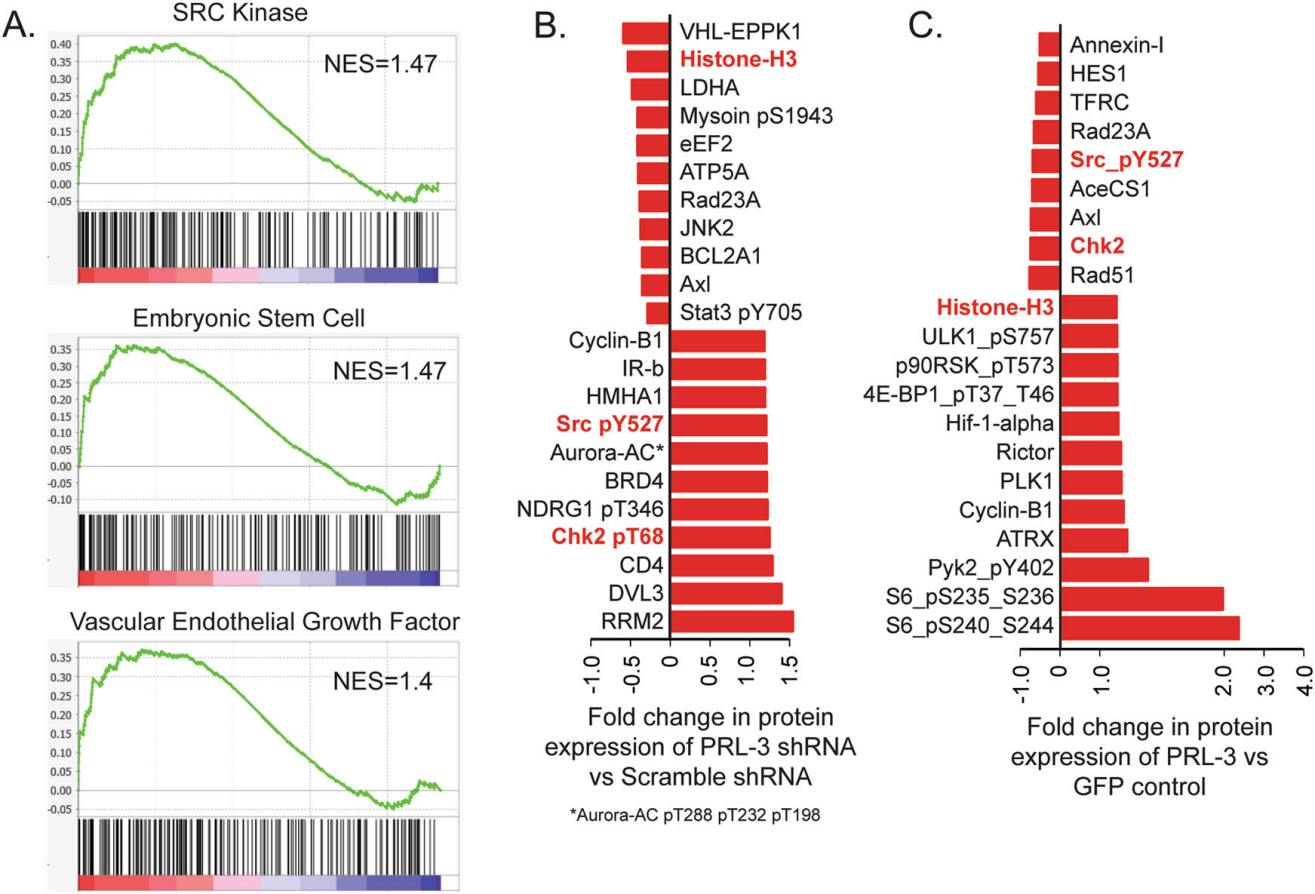
Src is a target of PRL-3. **a** GSEA analysis of T-ALL patients samples (GSE13159) comparing bone marrow with high PRL-3 expression (upper quartile) vs low PRL-3 expression (bottom quartile), showing the normalized enrichement score (NES). Reverse-phase protein array analysis (RPPA) of (**b**) PRL-3 knock-down or (**c**) overexpression of PRL-3 in Jurkat cells showed differential protein expression when compared to controls. Red bars show any protein that was up or down regulated 20%, and protein names shown in red are common in both groups, and include Chk2, Histone H3, and Src_pY527.

Our in vitro and in vivo data suggest that PRL-3 functions in T-ALL progression by modulating leukemia cell migration. To identify a mechanism by which PRL-3 might contribute to cell motility, we first examined gene signatures associated with PRL-3 expression in T-ALL patient samples. T-ALL samples with high levels of PRL-3 (upper quartile) and low levels of PRL-3 (lower quartile) were selected from GSE13159 (Fig. 1a) for Gene Set Enrichment Analysis (GSEA), which identified 24 pathways that were significantly different between the groups. Although PRL-3 was not associated with genes linked to any particular subtype of T-ALL, genes linked with SRC kinase signaling, an embryonic stem cell signature, and VEGF pathways were significantly enriched in PRL-3 high T-ALL (Fig. 4a and Supplemental Table 1). Additionally, Reverse-Phase Protein Array (RPPA) on 422 proteins and phospho-proteins identified ~20 proteins that showed differential expression between PRL-3 knock-down or PRL-3 overexpression T-ALL cell lines and the appropriate controls (Fig. 4b, c, Supplemental Tables 2,3). Top hits in both knock-down and overexpression cells included Histone-H3, Chk2, and Src_pY527.

**Fig. 5:**
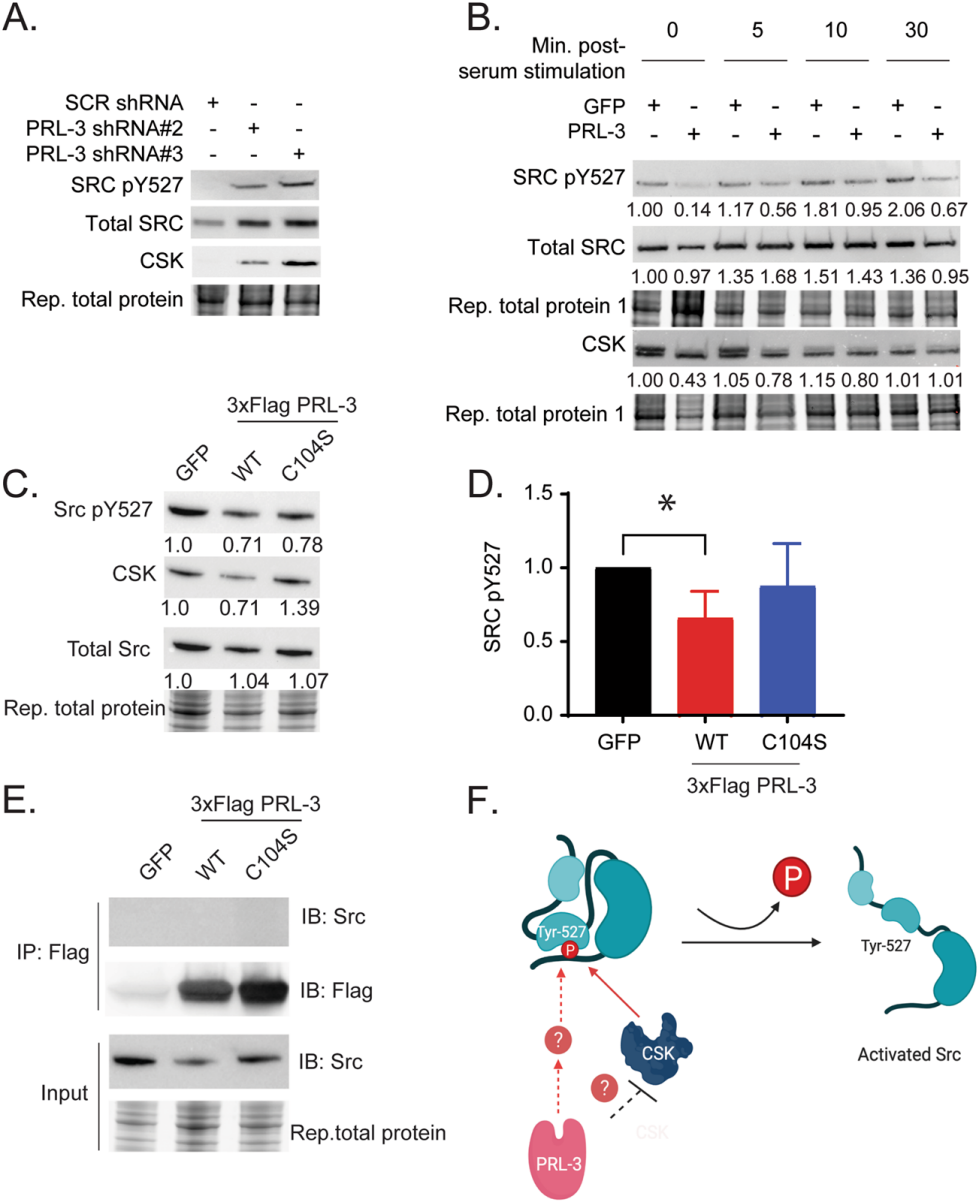
PRL-3 modulates Src phosphorylation. **a** Representative western blot analysis of Src_pY527, total Src, and CSK in Jurkat cells with PRL-3 knock-down. **b** Western blot validation of Src pathway in PRL-3 overexpressing Jurkat cells. Cells were serum starved overnight and added to serum containing complete media for the indicated time points. Numbers shown represent relative protein expression. **c** Representative western blot validation of Src pathway in 3xFlag PRL-3 Wt and 3xFlag PRL-3 C104S mutant Jurkat cells. **d** Quantification of *n* = 4 independent experiments analyzing SRC_pY527, *p = 0.003. **e** Co-immunoprecipitation assay of Jurkat overexpressing PRL-3 substrate trapping mutants, 3xFlag PRL-3 C104S or 3xFlag PRL-3 C104D, did not pulldown Src. **f** Schematic of PRL-3 and modulation of Src pathway in T-ALL cells.

Both GSEA and RPPA data suggest that the SRC pathway is associated with PRL-3 expression at both the mRNA and protein level. Src is a non-receptor kinase that is activated in a large fraction of cancers, where it plays a prominent role in cell migration and metastasis^37^. Src activity is negatively regulated by phosphorylation of tyrosine 527, which is an inhibitory phosphorylation site targeted by CSK (C-terminal Src Kinase). PRL-3 knock-down in Jurkat cells increased phosphorylation of Src_Y527 compared to scrambled shRNA control (Fig. 5a and Supplemental Fig. 6A), while PRL-3 overexpression decreased phosphorylation of Y527 (Fig. 5b and supplemental Fig. 6B). Interestingly, CSK expression was inversely correlated with PRL-3 expression (Fig. 5a, b), consistent in previous reports that found PRL-3 down-regulates CSK expression in human embryonic kidney cells and colon cancer cells^38^.

The effect of PRL-3 on phosphorylation of Src_Y527 may be through a direct action of PRL-3 phosphatase activity or through an indirect mechanism such as regulation of CSK or other proteins. We found that a phosphatase deficient mutant PRL-3, PRL-3(C104S) partially rescued the reduction of Src_Y527 phosphorylation compared to PRL-3 wild-type expression (Fig. 5c), suggesting that PRL-3 phosphatase activity likely plays a role in Src regulation. Importantly, PRL-3(C104S) has been previously shown to retain low levels of phosphatase activity^39^; the artificially high levels of exogenous PRL3(C104S) expression in the Jurkat cells may therefore compensate for reduced phosphatase activity, leading to incomplete rescue of the phosphorylation of Src_Y527.

Interestingly, we found that Src did not co-immunoprecipitate with Flag-tagged PRL-3 or PRL-3(C104S), despite being found at high levels in Jurkat cell lysate (Fig. 5e). These results indicate that Src is not a direct substrate of PRL-3 in T-ALL and PRL-3 modulates Src_Y527 phosphorylation by either inhibiting CSK expression, or via an unknown protein intermediate (Fig. 5f).

## Small molecule inhibition of PRL-3 reduces Src pathway activation and blocks T-ALL migration

**Fig. 6:**
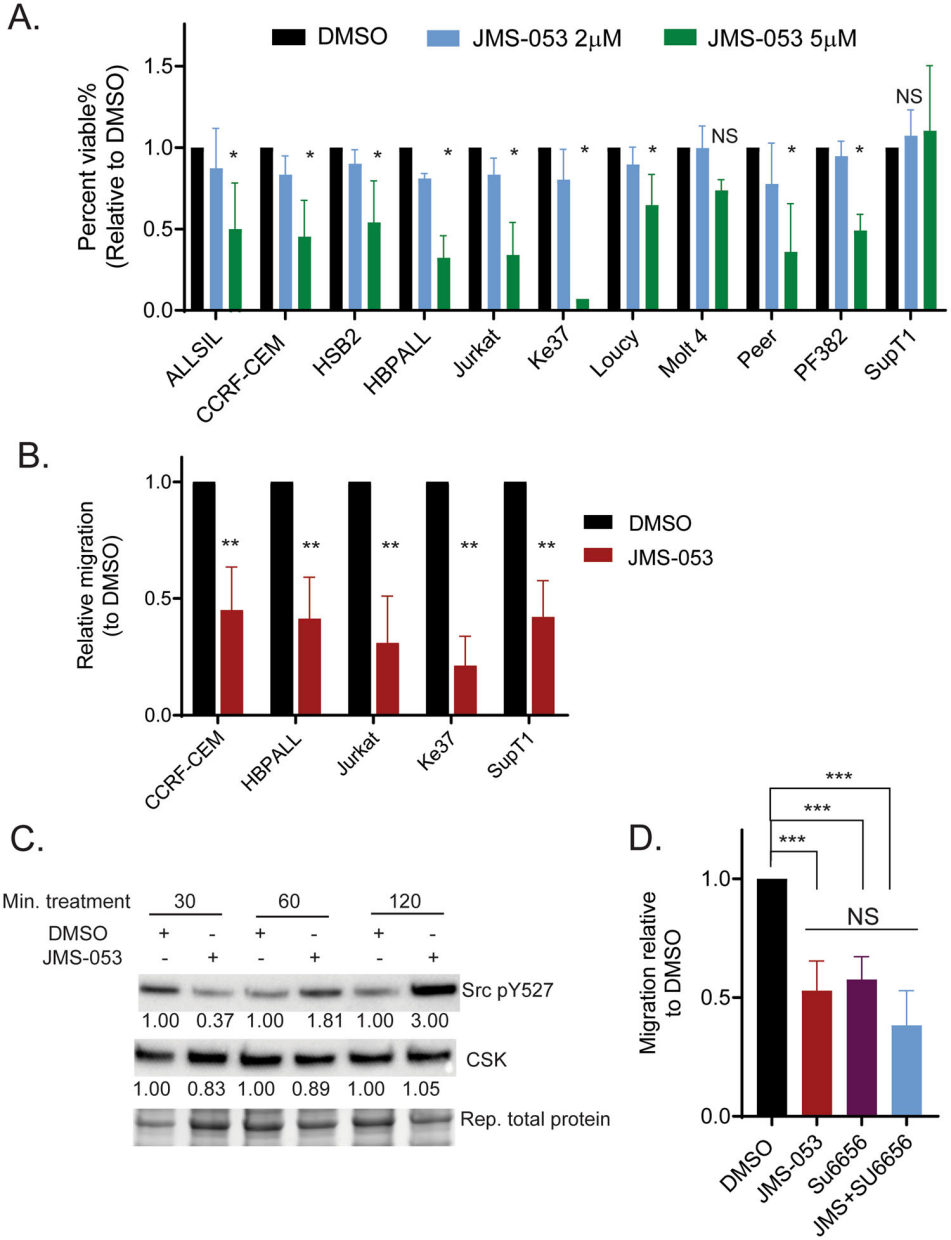
The PRL inhibitor JMS-053 reduces Src pathway activation and inhibits T-ALL migration. **a** JMS-053 reduced cell viability in T-ALL cell lines with high PRL-3 expression, evaluated by quantifying ATP production via Cell-Titer Glo, *p ≤ 0.001 or NS = not significant, compared to DMSO. **b** JMS-053 treatment (10 μM) for 2 h suppressed cell migration of T-ALL cells, **p < 0.001 compared to DMSO. For all, bars are the average of three experiments, each done in triplicate, ± standard deviation. **c** JMS-053 (10 µM) treatment increased Src phosphorylation at tyrosine 527. Blots are representative of at least three independent experiments. The numbers in the blot are relative expression normalized to total protein loaded. **d** Cell migration capability of PRL-3 overexpressing cells was compared between groups treated with DMSO, Src inhibitor Su6656 (2.5 μM), JMS-053 (10 μM) or in combination and showed no additive effects between Su6656 and JMS-053, NS = not significant, ***p < 0.05.

T-ALL migration plays a critical role in T-ALL progression and our data show PRL-3 can directly affect the migratory phenotype of T-ALL cells both in vitro and in vivo. Small molecule inhibition of PRL-3 can block solid tumor progression^40^, and we wanted to examine the effects of PRL-3 inhibition in T-ALL cells. The non-competitive small molecule PRL inhibitor, JMS-053^40^ significantly reduced the viability of T-ALL cells in a dose dependent manner (Fig. 6a), with lesser to no effects in cell lines that did not routinely express high levels of PRL-3. PRL-3 inhibition increased apoptosis in T-ALL cells after 24 h, although this trend was not significant across multiple experiments (Supplemental Fig. 7A), with no effect on cell cycle, measured by EdU uptake (Supplemental Fig. 7B). Short-term (<2 h) JMS-053 treatment significantly (*p* < 0.001) impaired the migration capability of all PRL-3 expressing T-ALL cell lines tested, reducing cell migration through a transwell towards a serum stimulus by 30–80% (Fig. 6b). JMS-053 treatment increased the phosphorylation Src_Y527 (Fig. 6c), again indicating that PRL-3 promotes cell migration by activation of Src.

Finally, we used the Src inhibitor Su6656 to treat PRL-3 overexpressing cells in combination with JMS-053, and evaluated whether the inhibitors synergized to affect cell migration capability. While both JMS-053 and Su6656 significantly decreased cell migration compared to control, we found no significant additive effect when both inhibitors were used (Fig. 6d), supporting the hypothesis that PRL-3 modulates Src signaling to promote cell migration. Taken together, our data showed that small molecule inhibition of PRL-3 can block T-ALL growth and migration in vitro, likely due to Src inhibition, and suggest that PRL-3 might be a useful target to control T-ALL progression.

## Discussion

Compared to other types of leukemia, T-ALL is urgently lacking immunotherapies or molecularly targeted therapies, which correlates with a worse prognosis for patients who fail traditional chemotherapy regimens. Central nervous system (CNS) infiltration by T-ALL and both CNS and bone marrow relapse remain critical clinical challenges, with survival rates of relapsed disease as low as 40%^41^. The identification and characterization of important drivers of T-ALL progression are needed for the design of novel, targeted therapeutics.

We found PRL-3 was highly expressed in T-ALL patient samples and cell lines, consistent with studies reporting PRL-3 upregulation in solid tumors^42^ and B-ALL^43^. Importantly, we used two different animal models to demonstrate an oncogenic role for PRL-3 in T-ALL, which, to the best of our knowledge, is the first in vivo study demonstrating this finding. In zebrafish, PRL-3 expression enhanced the spread of T-ALL cells from the thymus into surrounding tissues and promoted their rapid entry into circulation. In mouse xenograft, human T-ALL cells with silenced PRL-3 expression had diminished capacity to engraft and mice remained generally leukemia-free throughout the study. While we hypothesize the lack of T-ALL engraftment associated with PRL-3 knock-down may be attributed to a decreased ability of the leukemia cells to migrate and home to the bone marrow or thymus niche after xenograft, this needs to be confirmed experimentally. Given PRL-3 is established as enhancing viability and preventing apoptosis in other cancers^44^, PRL-3 might be playing additional roles in vivo that contribute to fitness of the T-ALL cells.

Overall, our data suggest that the primary role of PRL-3 in T-ALL is to promote cell migration, similar to its role in solid tumors. Recently, invasion and migration phenotypes have emerged as important factors in T-ALL progression and relapse. For example, CCR7, a known regulator of T-lymphocyte migration, is necessary and sufficient to drive infiltration of T-ALL cells into the CNS in a mouse model^45^ and inhibition of CXCR3, another lymphocyte migratory factor, significantly reduced leukemic infiltration into bone marrow, spleen and CNS^46^. Whether PRL-3 expression drives migration on its own or is associated with lymphocyte migratory signaling cascades is an area we are actively investigating. Nonetheless, there is a strong precedent for genes involved in migration, such as CCR7, CXCR3, and now PRL-3, as having critical roles in T-ALL progression.

We have demonstrated that PRL-3 modulates the SRC signaling pathway in T-ALL cell lines. Src activation has been reported in many types of human cancer, with a prominent role in regulating motility, migration, and metastasis^37,47,48^. PRL-3 has been previously reported to play a role in Src pathway activation in solid tumors and benign human cell lines^38,49^. Our study expands the role of PRL-3 in SRC signaling pathway modulation to include T-ALL, suggesting that PRL-3 might be a central regulator of the Src signaling network across multiple cancer types. However, our data showed that Src _Y527 is not a direct target of PRL-3 in T-ALL. It is more likely that PRL-3 affects CSK protein levels, which can directly phosphorylate Src_Y527, yet the exact mechanism of SRC activation requires further investigation. Additionally, we cannot exclude other possible PRL-3 effectors that could also directly or indirectly affect Src_pY527. Our RPPA analyses determined that the expression of other proteins were affected by PRL-3, including Histone-H3, Chk2, JNK, Hes1, Rictor, Axl, and Hif1-alpha, all of which play known roles in tumor progression, and may represent novel mechanisms by which PRL-3 promotes T-ALL.

In summary, our study expanded the oncogenic role of PRL-3 to T-ALL using both in vitro and in vivo assays. We found PRL-3 promotes T-ALL development and onset in both zebrafish and mouse xenograft models. Importantly, we also found that chemical inhibition of PRL-3 can inhibit cell growth and migration, suggesting that PRL-3 is a feasible therapeutic target in T-ALL. Cell-culture based assays revealed that PRL-3 modulates SRC signaling in T-ALL to enhance migratory capability, with no significant effect on cell growth. Given that several genes involved in T-cell migration promote CNS and bone marrow relapse, further studies on the role of PRL-3 in CNS infiltration and relapse of T-ALL are necessary. There is increasing interest in developing PRL-3 inhibitors for use in solid tumors; our study indicates that they may be useful in T-ALL as well.

## Materials and methods

### Antibodies, DNA plasmids, and other reagents

Antibodies used in this study, including their manufacturer, catalog number, lot number, blocking buffer used, and dilution factor are listed in Supplemental Table 4. The specificity of antibodies against PRL-1, 2, and 3 were validated against purified protein (Supplemental Fig. 8). The PRL inhibitor JMS-053 was kindly provided by Dr. John S. Lazo, Elizabeth Sharlow, and Peter Wipf (University of Virginia, Charlottesville, VA, USA), and the Src inhibitor, SU6656, was purchased from Sigma-Aldrich (S9692, St. Louis, MO, USA).

Lentiviral packaging plasmids psPAX2 (Addgene 12260, Watertown, MA, USA) and pMD2.G (Addgene 12259) were from Didier Trono. pLenti PGK Puro DEST (w529-2) (hereafter referred to as PGK) (Addgene 19068) and pLenti PGK GFP Puro (w509-5) (Addgene 19070) were from Eric Campeau & Paul Kaufman. Non-targeting control pLKO shRNA lentivirus plasmid (MISSION, SHC002, Sigma-Aldrich) was kindly provided by Tianyan Gao and pLKO shRNAs targeting PRL-3 were purchased from Sigma-Aldrich; target sequences are listed in Supplemental Table 5.

pENTR:*PRL-3* (human) and pENTR:*prl-3* (zebrafish) Gateway Entry constructs were made by PCR amplifying *PRL-3* and *prl-3* from cDNA generated from human T-ALL cells and 24 h post-fertilization zebrafish embryos, respectively. The PCR products were subcloned into the pENTR-d-TOPO cloning vector (ThermoFisher K2400-20, Waltham, MA, USA). The Gateway compatible zebrafish rag2 vector and generation of the *rag2:Myc* and *rag2:mCherry* construct has been previously described^34^. The *PGK:PRL-3* and *rag2:prl-3* constructs were generated using the PGK destination vector and *pENTR:PRL-3* or the rag2 destination vector and *pENTR:prl-3* along with Gateway LR Clonase II enzyme mix, according to manufacturer’s protocol (ThermoFisher 11791020).

### T-ALL cell lines and cell culture

All the human T-ALL cell lines used in the study were authenticated by short tandem repeat (STR) DNA profiling and tested for mycoplasma contamination prior to experimentation. Cells were grown in RPMI1640 (ThermoFisher 11875119) supplemented with 10% heat-inactivated fetal bovine serum (Atlanta Biologicals, S11150H, Lot M17161, Flowery Branch, GA, USA). Cells were cultured at 37 °C in a humidified atmosphere with 5% CO_2_.

### Western blot

Western blot analysis was performed using a stain-free technology developed by BioRad, which allows use of total protein as the loading control^50,51^. For all figures shown, the bands of total protein ~50 kD in size are used to represent the lane of total protein. An example of the entire lane that is used in the normalization calculations is shown in Supplemental Fig. 9.

### Co-immunoprecipitation

Cells (~50 million) were lysed in Pierce IP lysis buffer (Thermo 87788) supplemented with 1% Protease Inhibitor Cocktail (Sigma P8465). Total protein was incubated with 80 μL Anti-Flag M2 magnetic beads (Sigma m8823) on an orbital shaker overnight. After removing supernatant and washing the beads with PBS, the magnetic beads were boiled with 50 μL SDS containing buffer to elute the immunoprecipitants from the beads. Total cell lysates and immunoprecipitants were used for western blot analysis.

### Primary human samples

Frozen isolated PBMCs from de-identified T-ALL patients were kindly provided by Dr. Michelle Kelliher (University of Massachusetts Medical School, Worcester, MA, USA). PBMCs from healthy donors were purchased from Precision for Medicine (Bethesda, MD, USA).

### Microarray data analysis

The primary patient microarray datasets were accessed through the Gene Expression Ominbus (GEO) at NCBI (https://www.ncbi.nlm.nih.gov/geo), including GSE13159^52,53^ and GSE14615^54,55^. GSEA was done using GSEA 4.0.0. PRL-3 expression levels, corresponding to Affymetrix probes 209695_at and 206574_at were used for phenotypic labeling. Enrichment was calculated using MSigDB collection C6, oncogenic gene sets. Gene set enrichment was considered significant if it had a nominal *p*-value < 0.05.

### Lentivirus packaging and T-ALL cells infection

For PRL-3 knock-down, lentivirus was produced in 293T cells using TransIT-LT1 (Mirus Bio MR2300, Madison, WI, USA), according to the manufacturer’s instructions using scrambled or shPRL-3 plasmids. For T-ALL cell infection, 2.5 mL virus with 10 μg/mL polybrene (Thermo Fisher Scientific TR-1003-G) was added to 5 × 10^5^ cells and centrifuged at 2250 rpm for 90 min. Virus was washed out with PBS after 24 h, and cells were selected in culture media with 5 μg/mL puromycin for 48 h before experiments.

To generate PRL-3-ovexpressing cell lines, 293T cells were transfected with PGK:*GFP* with or without PGK:*PRL-3* or PGK:*3xFLAG-PRL-3* as described above. T-ALL cells were selected in medium with 5 μg/mL puromycin (Jurkat) or 1 μg/mL puromycin (HBP-ALL) for one week to produce stably expressing cell lines, then maintained in media with puromycin thereafter.

### In vitro cell-based assays

The CellTiter-Glo Luminescent Cell Viability Assay (Promega, G7570, Madison, WI, USA) was used to measure cell survival according to the manufacturer’s instructions. A Synergy LX BioTek (Winooski, VT, USA) multi-mode plate reader was used to read luminescent signal.

Migration assays were performed as previously described^56^. In experiments using JMS-053 or SU6656, cells were pre-treated with JMS-053, SU6656, or DMSO control for 2 h before plating into the upper chamber. The cells that migrated into the lower chamber were quantified by CellTiter-Glo Luminescent Cell Viability Assay.

Cell cycle was analyzed by quantifying 5′-ethynyl-2′-deoxyuridine (EdU) uptake using ClickIT EdU Alexa Fluor 647 (Thermo Fisher Scientific, C10424) according to the manufacturer’s protocol. DAPI (0.1 μg/ml) (ThermoFisher 62248) was used to stain the DNA.

Apoptosis was quantified by staining cells with Annexin V APC (ThermoFisher 88-8007-74) according to the manufacturers protocol, in the presence of DAPI (0.05 μg/ml)

### RPPA assay and data processing

Reverse Phase Protein Array (RPPA) and data analysis were performed by the RPPA Core Facility at MD Anderson Cancer Center (Houston, TX, USA) as previously described^57^.

### Zebrafish T-ALL models

Use of zebrafish was approved by the University of Kentucky’s Institutional Animal Care and Use Committee (IACUC), protocol 2015–2225. Microinjections of 15 ng/μL *rag2:Myc* *+* 45 ng/μL *rag2:mCherry* or 15 ng/μL *rag2:Myc* *+* 15 ng/μL *rag2:prl-3* *+* 30 ng/μL *rag2:mCherry* were used to generate zebrafish T-ALL in CG1 strain zebrafish as previously described, and number of animals used in each group were chosen based on previous experiments^35,58^. Zebrafish were monitored for leukemia onset and progression starting at 21 days post-fertilization (dpf) and every 3 days onwards by analyzing percent of the body expressing mCherry-positive leukemia cells using a Nikon fluorescence-equipped SMZ25 microscope. Circulating mCherry-positive T-ALL was noted by examining the vessels within the tail vasculature. Animals were monitored until 90 dpf or until they had to be sacrificed due to leukemia burden. Animals that died before the end of the monitoring period without leukemia progression outside the thymus were excluded.

Zebrafish leukemias were harvested and May-Grunwald Giemsa staining were performed as previously described before imaging on a BioTek Lionheart FX microscope^34,35^. To assess gene expression, RNA was isolated from the cells using Zymo Research Quick-RNA kit (R1054, Irvine, CA, USA). Total RNA was reverse transcribed (BioRad iSCRIPT, 1708891) and real time PCR performed using iTaq Universal SYBR Green Supermix (Biorad, 1725120) with primer sequences available in Supplemental Table 6. Data were normalized to ef1a expression and fold change was calculated using the 2^-∆∆^Cq method.

### Xenograft models in immune-compromised mice

Use of mice was approved by the University of Kentucky’s IACUC, protocol 2017–2754. Eight-week old NOD.Cg-Prkdc^*scid*^Il2rg^tm1Wjl^/SzJ (NSG) mice were obtained from Jackson Laboratory (Bar Harbor, ME, USA). Eight mice per group were used for experiments based on pilot studies that utilized three mice per group. The mice were randomized by placing into groups such that the difference between average group weight is not greater than 10%. Jurkat cells were infected with Scrambled shRNA or PRL-3 shRNA as described above. Two days after virus infection, Jurkat cells were selected using 5 μg/ml puromycin for two days, stained with trypan blue, and viable cells were FACS isolated. 10^6^ live cells in 100 μL PBS were injected intravenously. Peripheral blood samples (100–150 μL) were collected by submandibular bleeding at 4, 6, and 8 weeks post-transplantation and stained with human CD45 antibody according to Biolegend’s protocol and analyzed by flow cytometry.

### Statistical analysis

Results are shown as mean ± standard deviation. Sample sizes and number of replicates were chosen based on pilot experiments utilizing three samples per group, and experiments were done unblinded. At least three biological replicates were performed in each experiment. Statistical analyses were performed using GraphPad Prism 7 (San Diego, CA, USA), combining data from all samples across all replicates. Two-tailed *t*-tests were performed to compare two groups with similar distribution, and Analysis of Variance (ANOVA) with Tukey’s multiple comparisons was used to compare more than two groups. Human microarray data were analyzed using two-sample t-test and Wilcoxon rank sum tests, and survival curves were analyzed using Log-rank tests. All bar graphs shown are data pooled from ≥3 experiments.

## Supplementary information

Supplemental Figures

Supplemental Table 1 GSEA Analysis

Supplemental Table 2 RPPA Knockdown

Supplemental Table 3 RPPA Overexpression

Supplemental Table 4 Antibodies

Supplemental Table 5 shRNA sequences

Supplemental Table 6 RT PCR primers

Supplemental Video 1 PRL3+ circulating T-ALL

Supplemental Video 2 Myc non-circulating T-ALL
