# Supplementary material for: Protein tyrosine phosphatase 4A3 (PTP4A3/PRL-3) drives migration and progression of T-cell acute lymphoblastic leukemia in vitro and in vivo
Source: Oncogenesis. 2020 Jan 30;9(1):6. doi: 10.1038/s41389-020-0192-5 (PMC6992623; doi:10.1038/s41389-020-0192-5)
Supplement: Supplementary file 1 — Supplemental Figures [file 41389_2020_192_MOESM1_ESM.pdf]

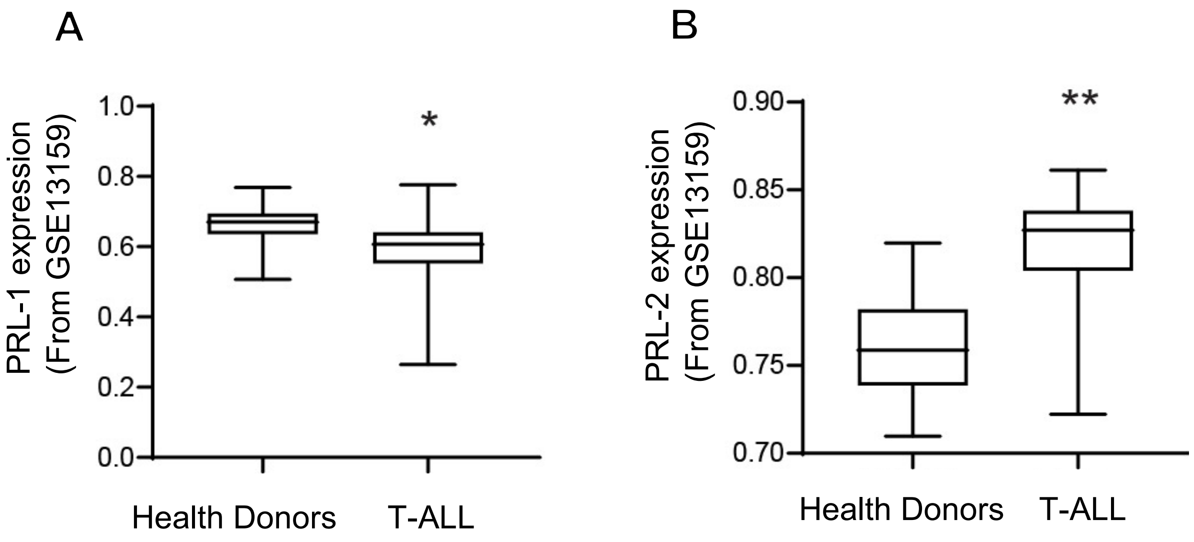

Supplemental Figure 1. Expression of PRL-1 and PRL-2 in T-ALL. Microarray expression analysis of PRL-1 (A) and PRL-2 (B) expression in bone marrow samples from T-ALL patient compared to that from healthy donors. **p*<0.001 PRL-1 in T-ALL compared to PBMCs, ***p*<0.0001 PRL-2 expression in T-ALL compared to PMBNC.

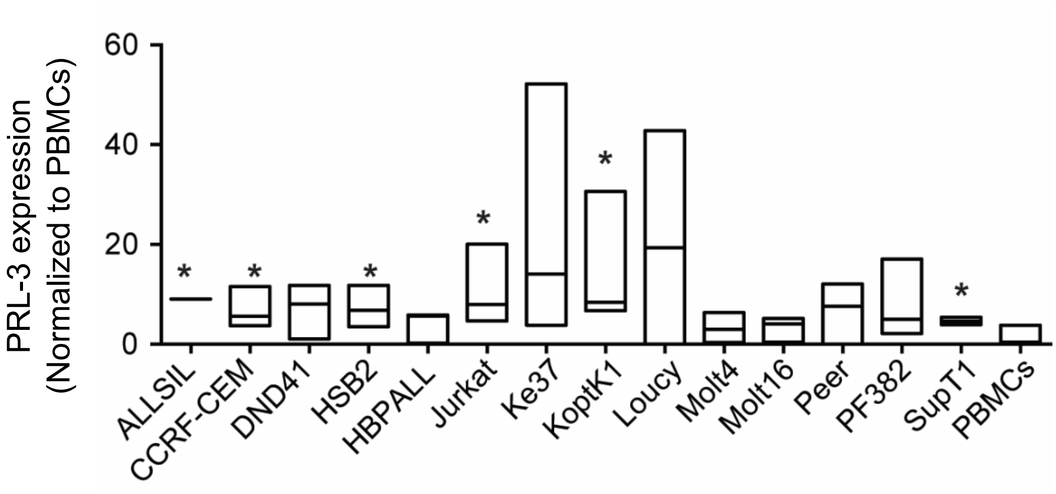

Supplemental Figure 2. Variation of PRL-3 expression in T-ALL cells. Quantification of 3 independent western blots cross human T-ALL cell lines, showing that PRL-3 expression can vary significantly, even within the same cell line. *p<0.001 PRL-3 in T-ALL compared to PBMCs.

A.

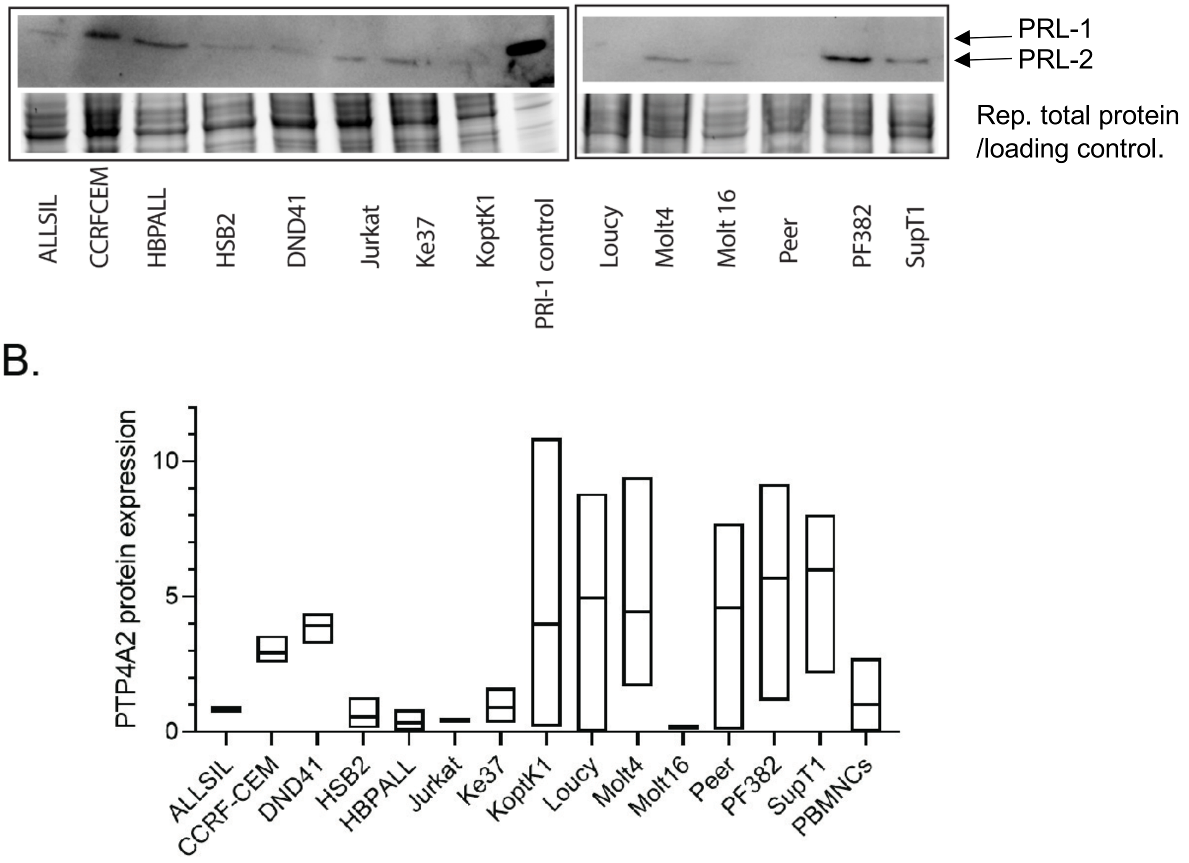

Supplemental Figure 3. T-ALL cells lines express PRL-2 but not PRL-1. (A) Representative western blot analysis of PRL-1 and PRL-2 in human T-ALL cell lines, showing PRL-2 expression in a subset of T-ALL cell lines. Representative bands from total protein loaded was used for loading control (B) Quantification of 3 independent western blots cross human T-ALL cell lines, showing that PRL-2 expression can vary, even within the same cell line.

A.

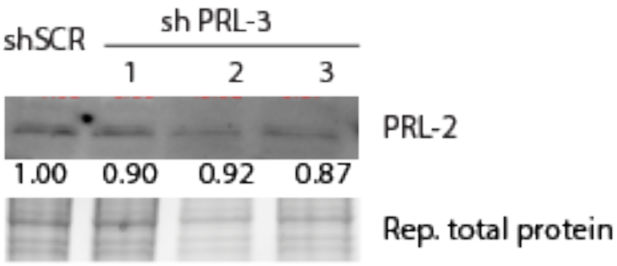

B.

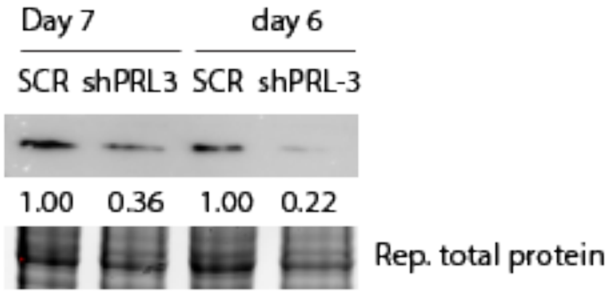

Supplemental Figure 4. Effects of PRL-3 knock-down. Western blot analysis shows PRL-2 expression does not change upon PRL-3 knock-down, indicating PRL-2 does not compensate for PRL-3 loss. (B) Jurkat cells, which showed PRL-3 complete knock-down at day 5 post lentiviral infection (Figure 3) were cultured for 6 and 7 days and total protein was extracted for western blot analysis, showing that PRL-3 expression returns over time. Blots are representative of at least 3 independent experiments. The numbers in the blot are relative expression normalized to total protein loaded.

A.

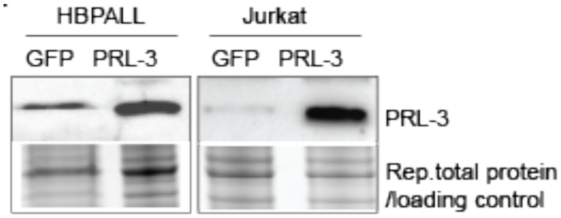

B.

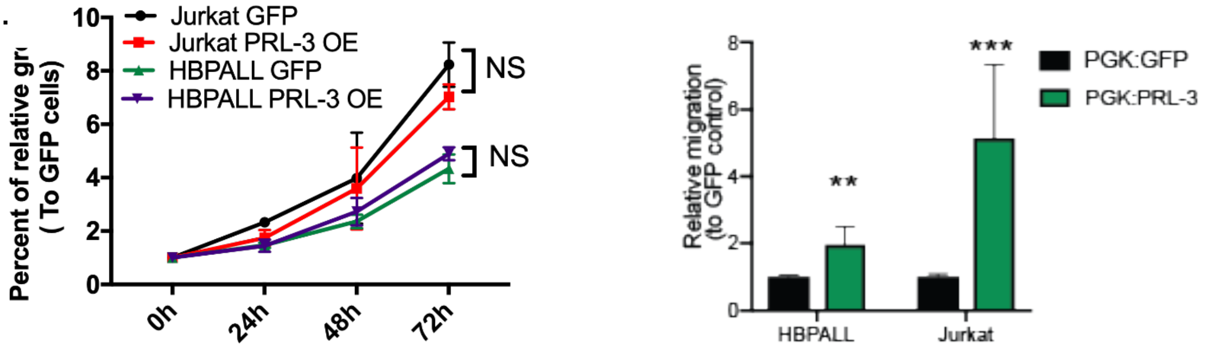

Supplemental Figure 5. PRL-3 promotes cell migration, but not growth, in T-ALL. (A) Western blot showed PRL-3 overexpression in HBPALL and Jurkat cells. (B) T-ALL cells expressing PGK:PRL-3 (PRL-3 OE) or PGK:GFP (GFP) were cultured in medium with puromycin for 72 hours. Cell growth was determined by Cell Titer-glow assay and normalized to the readout of day 0, and showed no difference between PRL-3 overexpressing cells and control cells at all timepoints. Data shown are the average of 3 independent experiments, done in triplicate, NS= not significant. (C) High PRL-3 expression increased cell migration in T-ALL cells. Migration was normalized to control cells expressing GFP only, **p<0.001, ***p<0.0001. For all bar graphs, data are the average of at least three independent experiments, done in triplicate.

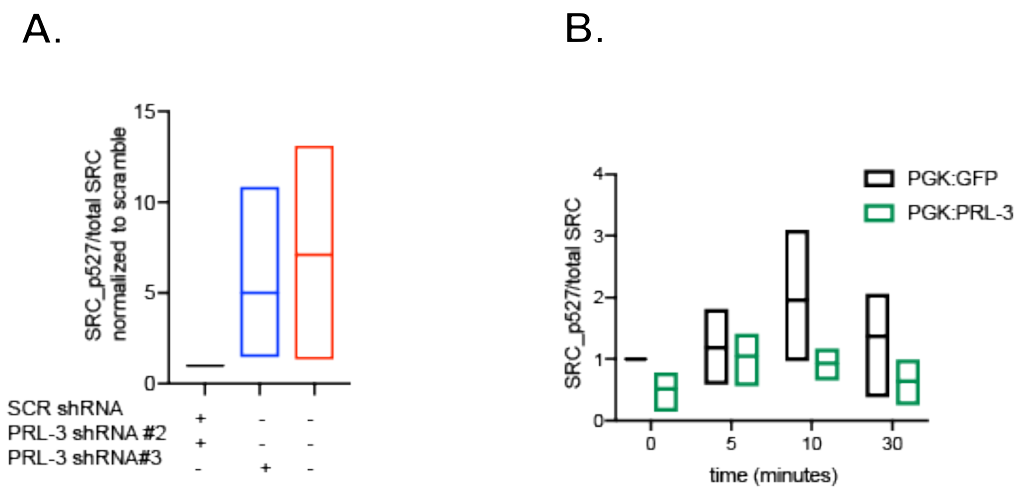

Supplemental Figure 6. Quantification of 3 independent western blots to show Src pY527 in PRL-3 Knock-down (A) or overexpressing (B) Jurkat cells.

A. B.

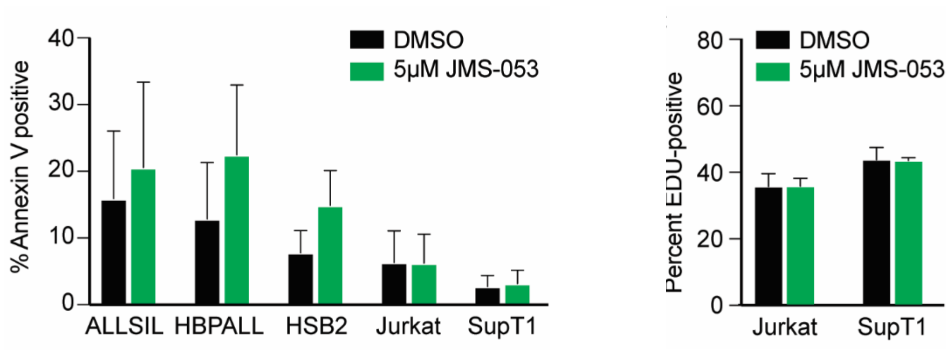

Supplemental Figure 7. The specific pan-PRL inhibitor JMS-053 does not affect apoptosis or cell cycle in T-ALL cells. T-ALL cells were treated for 24 hours with JMS-053 and (A) apoptosis was assessed by AnnexinV staining. (B) Cell proliferation was assessed based on Edu uptake. Cell staining was quantified by flow cytometry, and no significant difference was noted across 3 independent experiments, done in triplicate.

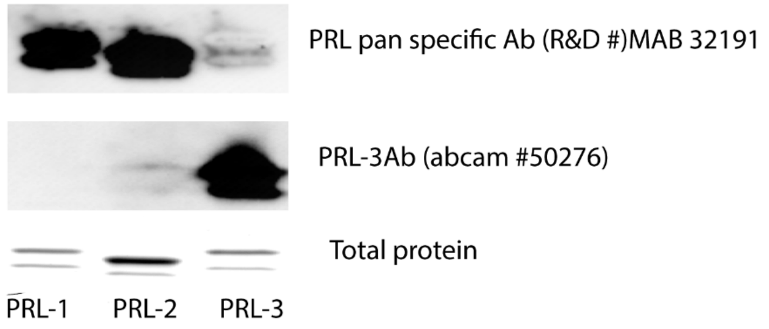

Supplemental Figure 8. PRL antibody validation. Antibody against PRL-3 (Abcam, 50276) detects purified PRL-3 protein specifically but not PRL-1 and 2. Human PRL pan specific antibody (MAB32191) detects both PRL-1 and PRL-2 at similar sensitivity but barely detects PRL-3. Recombinant human PRL-1 (8490-PT), PRL-2 (6694-PT) and PRL-3 (8455-PT) proteins were from R&D systems and 0.3 μg of each protein was loaded in 4-20% protein TGX stain-free gel (BioRad, cat #4568093).

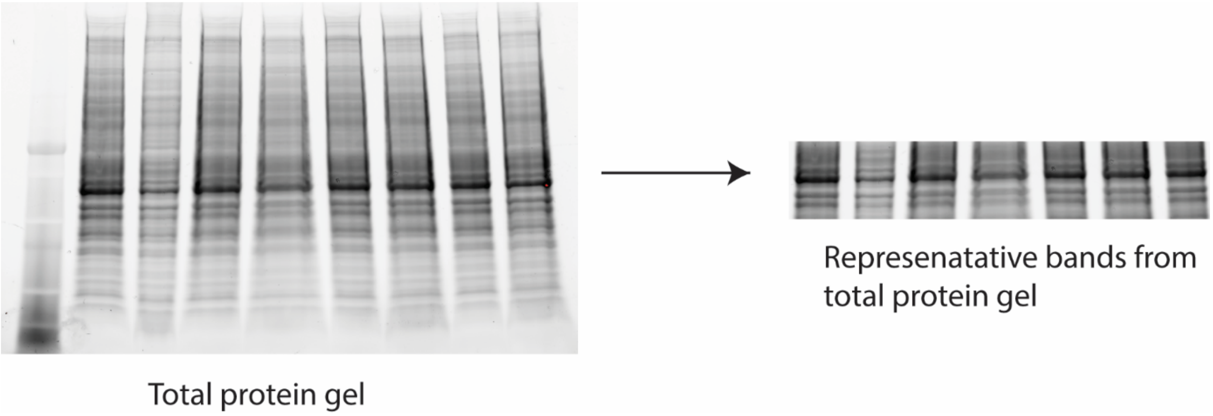

Supplemental Figure 9. Total protein as loading control for Western blot. All the Western blot analyses were performed using a stain-free technology developed by BioRad, which utilizes total protein loaded for each sample as the loading control. Protein was separated on TGX-stain free pre-casted 4-20% SDS gel, which allows the image of total protein, as shown in left, by exposing the gel under the UV activation in the ChemiDoc Imaging System. Representative bands from the total protein gel instead of the whole image of the gel was shown in the Western blots results in order to save space.
