# Supplementary material for: Protein tyrosine phosphatase 4A3 (PTP4A3/PRL-3) drives migration and progression of T-cell acute lymphoblastic leukemia in vitro and in vivo
Source: Oncogenesis. 2020 Jan 30;9(1):6. doi: 10.1038/s41389-020-0192-5 (PMC6992623; doi:10.1038/s41389-020-0192-5)
Supplement: Supplementary file 2 — Supplemental Table 1 GSEA Analysis [file 41389_2020_192_MOESM2_ESM.pdf]

Table S1: GSEA report of pathways signifiacntly up or down regulated in high PRL-3 expressing patient-derived T-ALL samples, compared to low PRL-3 expressing T-ALL

NAME	GS<br>follow link to MSigDB	SIZE	ES	NES	NOM p-val	FDR q-val	FWER p-val
PRC2_EED_UP.V1_DN	PRC2_EED_UP.V1_DN	184	0.4719	1.7851	0.0022	0.2004	0.093
GLI1_UP.V1_DN	GLI1_UP.V1_DN	23	0.5564	1.6973	0.0240	0.2343	0.179
SIRNA_EIF4GI_UP	SIRNA_EIF4GI_UP	88	0.3538	1.6392	0.0269	0.2726	0.279
PDGF_ERK_DN.V1_DN	PDGF_ERK_DN.V1_DN	139	0.3631	1.6155	0.0159	0.2479	0.321
BCAT_BILD_ET_AL_UP	BCAT_BILD_ET_AL_UP	44	0.4788	1.5861	0.0193	0.2507	0.373
CYCLIN_D1_UP.V1_DN	CYCLIN_D1_UP.V1_DN	181	0.3539	1.5588	0.0060	0.2247	0.435
CYCLIN_D1_KE_.V1_UP	CYCLIN_D1_KE_.V1_UP	180	0.3597	1.5346	0.0122	0.2359	0.476
ERB2_UP.V1_UP	ERB2_UP.V1_UP	181	0.3872	1.5205	0.0059	0.2343	0.51
CYCLIN_D1_UP.V1_UP	CYCLIN_D1_UP.V1_UP	178	0.3760	1.4965	0.0164	0.2578	0.568
CSR_LATE_UP.V1_DN	CSR_LATE_UP.V1_DN	158	0.3730	1.4812	0.0205	0.2619	0.599
MTOR_UP.V1_UP	MTOR_UP.V1_UP	151	0.3708	1.4737	0.0290	0.2538	0.613
SRC_UP.V1_UP	SRC_UP.V1_UP	162	0.4001	1.4727	0.0144	0.2363	0.616
ESC_J1_UP_EARLY.V1_UP	ESC_J1_UP_EARLY.V1_UP	165	0.3623	1.4684	0.0122	0.2270	0.623
RB_DN.V1_DN	RB_DN.V1_DN	117	0.3489	1.4496	0.0361	0.2451	0.671
RAPA_EARLY_UP.V1_DN	RAPA_EARLY_UP.V1_DN	176	0.3430	1.4385	0.0274	0.2328	0.689
ESC_V6.5_UP_EARLY.V1_UP	ESC_V6.5_UP_EARLY.V1_UP	158	0.3267	1.4236	0.0120	0.2441	0.712
MEK_UP.V1_UP	MEK_UP.V1_UP	188	0.3767	1.4152	0.0276	0.2441	0.728
VEGF_A_UP.V1_UP	VEGF_A_UP.V1_UP	183	0.3702	1.3953	0.0365	0.2685	0.759
KRAS.AMP.LUNG_UP.V1_DN	KRAS.AMP.LUNG_UP.V1_DN	135	0.3801	1.3773	0.0202	0.2879	0.779
CRX_NRL_DN.V1_UP	CRX_NRL_DN.V1_UP	127	0.3487	1.3708	0.0333	0.2869	0.785
GCNP_SHH_UP_EARLY.V1_DN	GCNP_SHH_UP_EARLY.V1_DN	161	0.3075	1.3629	0.0377	0.2895	0.796
KRAS.DF.V1_UP	KRAS.DF.V1_UP	187	0.3269	1.3556	0.0304	0.2945	0.813
IL2_UP.V1_DN	IL2_UP.V1_DN	181	0.3450	1.3160	0.0465	0.3436	0.869
E2F3_UP.V1_DN	E2F3_UP.V1_DN	142	0.3197	1.2844	0.0359	0.3900	0.899

down-regulated

up-regulated
