# Supplementary material for: Protein tyrosine phosphatase 4A3 (PTP4A3/PRL-3) drives migration and progression of T-cell acute lymphoblastic leukemia in vitro and in vivo
Source: Oncogenesis. 2020 Jan 30;9(1):6. doi: 10.1038/s41389-020-0192-5 (PMC6992623; doi:10.1038/s41389-020-0192-5)
Supplement: Supplementary file 5 — Supplemental Table 4 Antibodies [file 41389_2020_192_MOESM5_ESM.pdf]

Table S4: List of antibodies, their source and working dilutions

Reagents	Source	Host species	Working dilution
Anti-PTP4A3	Abcam (ab50276, Lot GR9977-20)	Rabbit	1:500
Anti-PTP4A	R&D(MAB32191, Lot XJS01)	Mouse	1:1000
Anti-Src	Cell Signaling (Clone 32G6, 2123, Lot 5)	Rabbit	1:1000
Anti-Src_pY416	Cell Signaling (Clone D49G4, 6943, Lot 4)	Rabbit	1:1000
Anti-Src_pY527	Cell Signaling (2105, Lot 9)	Rabbit	1:1000
Anti-CSK	Cell Signaling (Clone C74C1,4980, Lot 2)	Rabbit	1:1000
PE Anti-Human CD45	Biolegend (Clone HI30, 304008)	Mouse	1:1000
Mouse IgG-HRP	Cell Signaling (7106, Lot TC2625)	Goat	1:5000
Rabbit IgG-HRP	GeneTex (26741, Lot 9788061)	Goat	1:5000
Blocking buffer	5% milk in 1% TBST		
