# Supplementary material for: Protein tyrosine phosphatase 4A3 (PTP4A3/PRL-3) drives migration and progression of T-cell acute lymphoblastic leukemia in vitro and in vivo
Source: Oncogenesis. 2020 Jan 30;9(1):6. doi: 10.1038/s41389-020-0192-5 (PMC6992623; doi:10.1038/s41389-020-0192-5)
Supplement: Supplementary file 6 — Supplemental Table 5 shRNA sequences [file 41389_2020_192_MOESM6_ESM.pdf]

Table S5: shRNA sequence used for PTP4A3 knock-down

	targeted sequence
PTP4A3 shRNA #1	CTACAAACACATGCGCTTCCT
PTP4A3 shRNA #2	TCTCGGCACCTTAAATTATTA
PTP4A3 shRNA #3	ACCGTTGTGGACTGGCCGTTT
