# Supplementary material for: Protein tyrosine phosphatase 4A3 (PTP4A3/PRL-3) drives migration and progression of T-cell acute lymphoblastic leukemia in vitro and in vivo
Source: Oncogenesis. 2020 Jan 30;9(1):6. doi: 10.1038/s41389-020-0192-5 (PMC6992623; doi:10.1038/s41389-020-0192-5)
Supplement: Supplementary file 7 — Supplemental Table 6 RT PCR primers [file 41389_2020_192_MOESM7_ESM.pdf]

Table S6. Realtime RT-PCR primer sequences

Gene	Forward Primer	Reverse Primer	Purpose
z-ptp4a3	GGTGTCACGACAGTGGTCAG	TCAATCAAGGCCACAGCCAC	
z-rag1	AGCAATGATGCAAGGCAGAG	TGTGCAGGGGCTGGAATATC	Lymphocytes
z-rag2	AGCTCTCAGATTTCGGAGTACAC	ACAAGGCTGCCACAATTCAC	Lymphocytes
z-lck	AGAAGATCTCGATGGTTTGTCTGT	CGCAGTTCCCCATGTTTACG	T-cell
z-tcr β-c2	ATTCACCTGCACTGTCCGAT	AGCTTCAATCCCTTCGGCTT	T-cell
z-pax5	CTGATTACAAACGCCAAAAC	CTAAATTATGCGCAGAAACG	B-cell
z-igD	GAGAGCAGCAAAAGGATGGC	TGCAAGTTTGGTCTTGTTCTGC	B-cell
z-IgM-VH1	CATGACAATGGATATTGTGTCC	ACATGAAGGTTGCTGATCCAC	B-cell
m-cMyc	AGCGACTCTGAAGAAGAGCAA	GCACCTCTTGAGGACCAGTG	
z-eef1a1	ATGGCACGGTGACAACATGCT	CCACATTACCACGACGGATG	normalizing
